# UET: a database of evolutionarily-predicted functional determinants of protein sequences that cluster as functional sites in protein structures

**DOI:** 10.1093/nar/gkv1279

**Published:** 2015-11-20

**Authors:** Rhonald C. Lua, Stephen J. Wilson, Daniel M. Konecki, Angela D. Wilkins, Eric Venner, Daniel H. Morgan, Olivier Lichtarge

**Affiliations:** 1 Department of Molecular and Human Genetics, Baylor College of Medicine, Houston, TX 77030, USA; 2 Department of Biochemistry and Molecular Biology, Baylor College of Medicine, Houston, TX 77030, USA; 3 Department of Structural and Computational Biology and Molecular Biophysics, Houston, TX 77030, USA; 4 Computational and Integrative Biomedical Research Center, Baylor College of Medicine, Houston, TX 77030, USA; 5 Department of Pharmacology, Baylor College of Medicine, Houston, TX 77030, USA

## Abstract

The structure and function of proteins underlie most aspects of biology and their mutational perturbations often cause disease. To identify the molecular determinants of function as well as targets for drugs, it is central to characterize the important residues and how they cluster to form functional sites. The Evolutionary Trace (ET) achieves this by ranking the functional and structural importance of the protein sequence positions. ET uses evolutionary distances to estimate functional distances and correlates genotype variations with those in the fitness phenotype. Thus, ET ranks are worse for sequence positions that vary among evolutionarily closer homologs but better for positions that vary mostly among distant homologs. This approach identifies functional determinants, predicts function, guides the mutational redesign of functional and allosteric specificity, and interprets the action of coding sequence variations in proteins, people and populations. Now, the UET database offers pre-computed ET analyses for the protein structure databank, and on-the-fly analysis of any protein sequence. A web interface retrieves ET rankings of sequence positions and maps results to a structure to identify functionally important regions. This UET database integrates several ways of viewing the results on the protein sequence or structure and can be found at http://mammoth.bcm.tmc.edu/uet/.

## INTRODUCTION

The Evolutionary Trace (1,2) (ET) was developed as a scalable computational method to identify functionally and structurally important sequence positions. In turn, knowing the molecular determinants of protein structure and function has many critical applications across biology and medicine. For example, to guide efficient mutagenesis (3,4); interpret patient mutations (5–7); design potential therapeutic peptides (8–10); engineer separation of function in animal models (11); extract functional motifs that predict functions and substrates over the structural proteome (12–14); and measure the molecular, clinical and population-wide action of human coding variations (5,15).

ET uses the ‘evolutionary record,’ to establish a relative rank among sequence positions. Those positions that vary mostly among distant homologs rank ahead of positions that vary mostly among evolutionarily close homologs. Critically, top-ranked ET residues consistently exhibit useful structural and functional features: they form statistically significant clusters in native protein structures (16), they overlap extensively with known functional sites (17) and they guide mutational studies that predictably alter function as well as form general 3D functional motifs (14).

Previous public tools for ET analysis of sequence, structure and function included, first, a Java ET Viewer (18), followed by the ET report_maker (19), JEvTrace (20), TraceSuite II (21) and PyETV (22). Both the Java ET Viewer and JEvTrace combine an interactive molecular view of the structure with the multiple sequence alignment and phylogenetic tree. TraceSuite II compiles the trace results together with snapshots of the structure, sequence alignment and tree in a webpage. The report_maker presents ET analysis superimposed on information about sequence, structure and elementary annotation, in a human-readable static PDF document. PyETV is an ET analysis plugin for the PyMOL molecular visualization platform (The PyMOL Molecular Graphics System, Version 1.7.4 Schrödinger, LLC.). However, these tools are now at least 5 years old. Modern platforms along with additional data demand an update for usability and wider applications. Moreover, some tools, like the combined PyMOL and PyETV methods of viewing ET information are more technical; they may require significant computer knowledge to implement and understand.

To facilitate access and broad use of ET analysis, we now present a new website and database called UET (Universal Evolutionary Trace). This is a repository of pre-computed ET analyses performed on the protein structure databank (PDB) (23). It can be accessed via a web interface using a given protein structure to retrieve an ET ranking of sequence positions and to identify functionally important regions in that structure. In UET, seamless integration of structure and phylogenetic tree viewers in the web browser means that a user can examine protein structures and sequences with their ET analyses, without any prior software installation (assuming the user's computer has a web browser). It also avoids access, update and digital signing issues that often plague viewers based on Java applets that run on browsers. Furthermore, tight integration with a web browser enables ET analysis to be accessible to ubiquitous mobile devices such as tablets and smart phones.

## FEATURES

### Inputs

UET stores ET analyses of unique protein chains in a PDB entry. In order to retrieve pre-computed ET analyses, the user is prompted for a PDB code plus a chain identifier (e.g. 2qrvA).

UET accepts several other inputs for *de novo* ET analysis. This significantly expands the coverage of ET analyses to include protein sequences without representative structures in the PDB, or structures that are custom produced (such as models). To be clear, these inputs may consist of a protein sequence (specified by a UniProt (24) accession number or explicitly in FASTA format) or of a novel structure (PDB coordinates file supplied by the user, in confidentiality). Of note, the user can also tailor the multiple sequence alignment and other parameters of the ET analysis.

### Structure view

**Figure 1.**
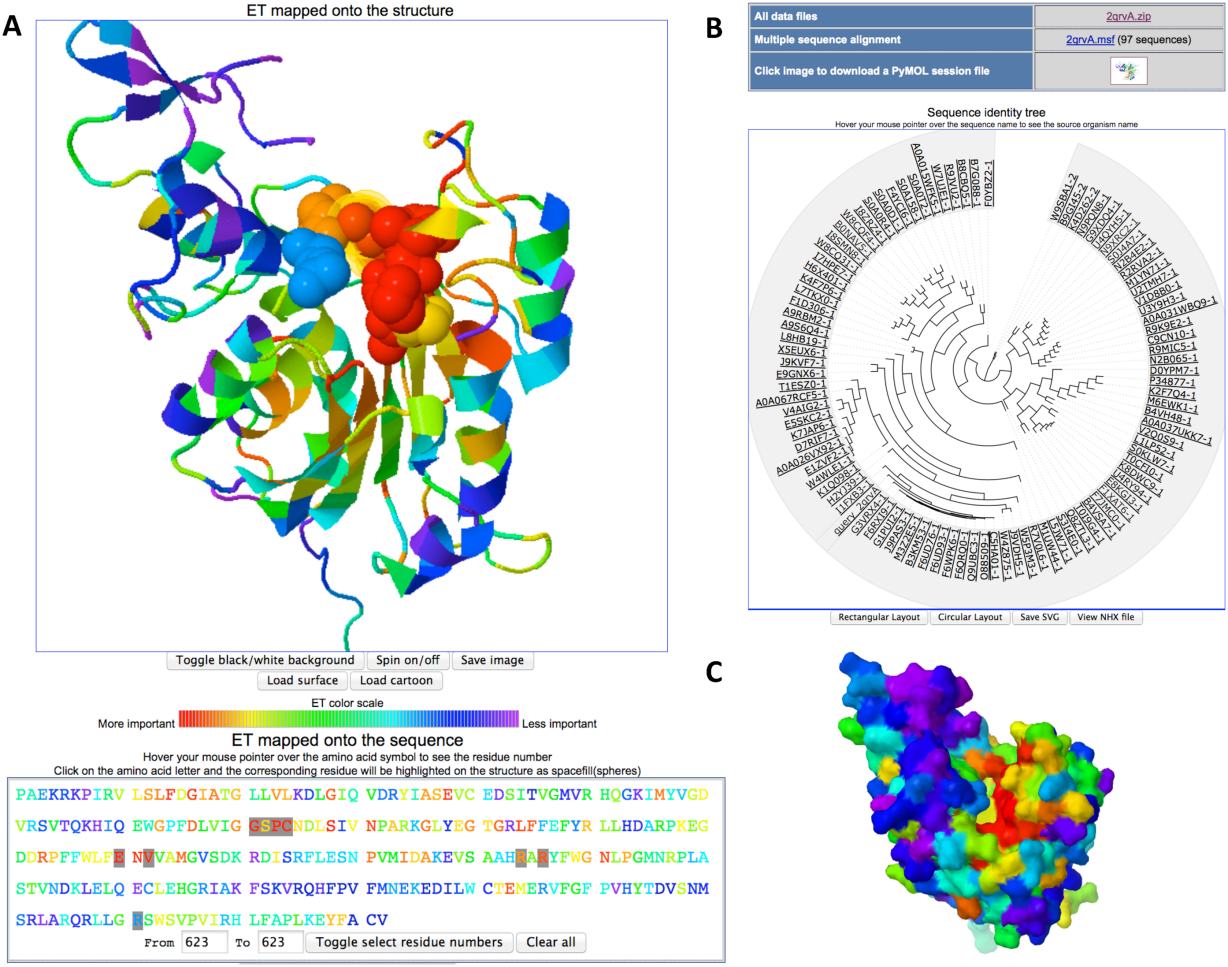
Example UET web browser output. (**A**) ET analysis of the DNA-binding domain of mouse DNMT3A (PDB code + chain identifier 2qrvA) (30) can be seen in the structure view with the DNA-binding site selected via the sequence view. Residues highlighted were within four Angstroms of the cytosine targeted by methylation (identified from superposition of PDB 1MHT (37) ). (**B**) Sequence identity tree view and links to data files. (**C**) When the surface view is selected, a surface rendering is visible that can help highlight important surface regions, such as binding sites.

To identify functional sites, the structure view shows a cartoon representation of the PDB structure, with prismatic colors that indicate the relative evolutionary importance of each residue according to its ET percentile rank (Figure 1A, red is most important and magenta is least so). The structure view exploits JSmol (25). The structure can be examined and manipulated in the usual intuitive way (left-mouse-click or double tap on a touchscreen, then drag to rotate, etc.). Placing the mouse-pointer over a residue will show its amino acid type and sequence number. The ‘Load surface’ option displays the protein surface making it easier to spot functional or binding sites (Figure 1C). ‘Save image’ lets the user save the current view of the structure into an image file. A right-mouse-click on the viewer reveals the JSmol menu with more visualization options.

### Sequence view

To promptly find the most evolutionarily important residues, the sequence view presents the chain of amino acids in one-letter code. As before, the color key indicates relative evolutionary importance according to the ET percentile rank of each position (Figure 1A).

The sequence view is coupled to the structure view. Selecting an amino acid letter code by a click of the mouse (or tap on the touchscreen) causes the corresponding residue in the structure to be highlighted with a spacefill representation of the residue. An option to select a series of residues at once is also available.

### Sequence identity tree view

As a guide in assessing the specificity and applicability of the predicted functional sites, the sequence identity tree used in the ET analysis is shown in a circular layout (the default, which can be switched to a rectangular layout)(Figure 1B). The tree view is provided by jsPhyloSVG (26), enhanced by a description of tree nodes using phyloXML (27). Hovering the mouse pointer over the sequence name will show the associated source organism or species. The tree view may also be saved as an SVG file, as well as in the raw tree data NHX format.

### ET analysis output data files

The ET analysis data files, including files necessary to view the results in PyMOL, can be downloaded through a link on the web output. The ET analysis pipeline is described elsewhere (28).

### Documentation

Multiple videos are online and show how to carry out an ET analysis with UET and with other tools. The URL is at http://www.youtube.com/user/EvolutionaryTrace.

## EXAMPLES

**Figure 2.**
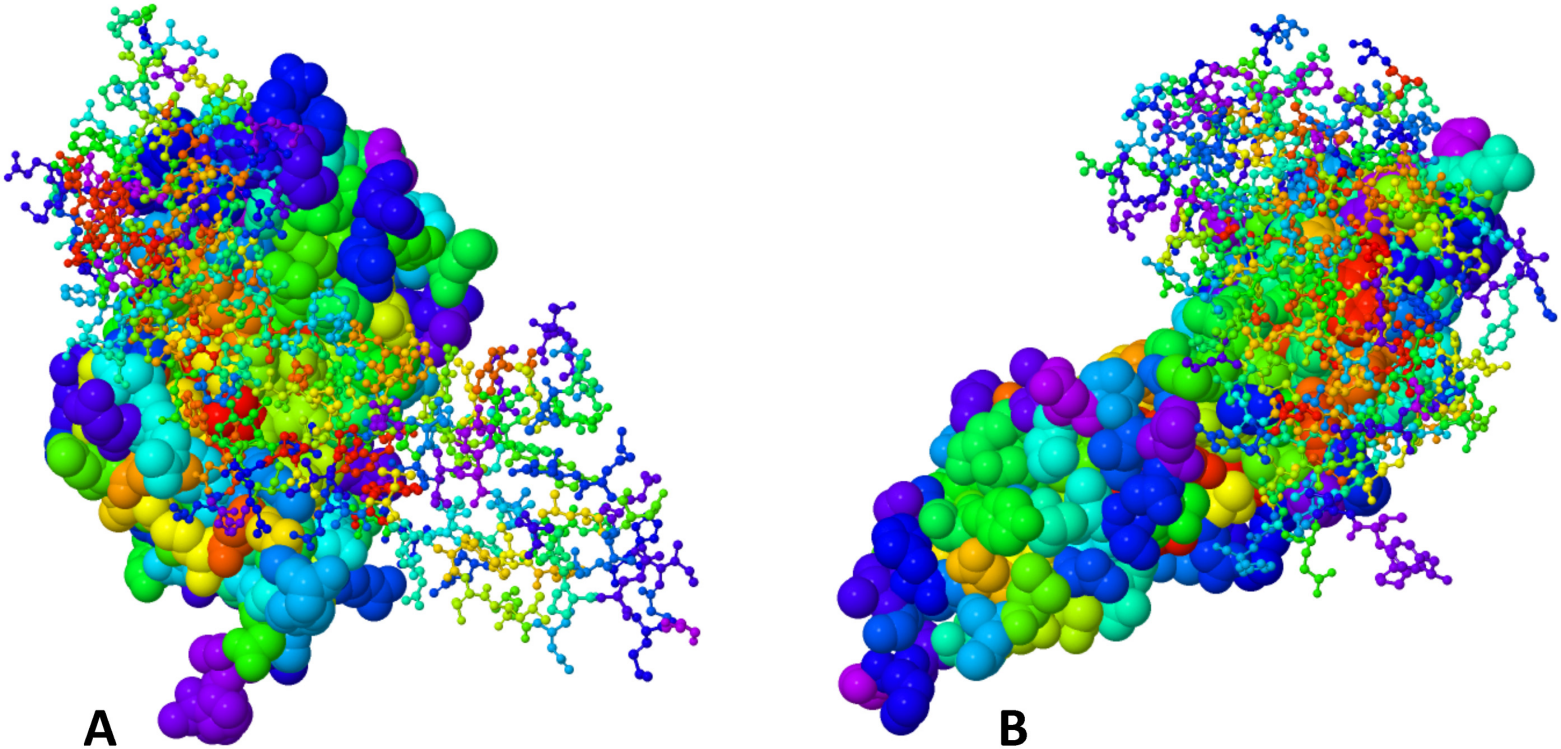
The human growth hormone in complex with the growth hormone receptor (PDB code: 1a22 (31)) with ET analysis. (**A**) Human growth hormone is shown in spacefill mode, while the human growth hormone receptor is shown as ball and stick. (**B**) The human growth hormone receptor is shown as a spacefill, while the human growth hormone is displayed as ball and stick.

ET has been extensively tested both in case studies and on a large scale. It identifies statistically significant clusters and functional sites within protein structures (16), and it guides the redesign of functional and allosteric sites (29). Such analyses often lead to new insights, now made more readily accessible with the release of the UET database and website interface. For example, UET of the DNA-binding domain of the DNA methyltransferase DNMT3A (PDB ID: 2qrv chain A) (30) from mouse, reveals a cluster of critically important residues immediately adjacent (4 Angstroms) to the cytosine targeted by methylation (Figure 1A). The most important residues are highlighted in red in the structure image and in the sequence mapping. Selecting these residues in the cartoon view shows that they tend to be central to the molecule, while switching to the surface view (Figure 1C) makes it apparent that they highlight a functional site. Likewise, clusters of evolutionarily important residues map the binding site between the human growth hormone and its receptor (PDB ID: 1a22 chains A and B, Figure 2) (31). Of note, ET performance can sensitively depend on the choice of parameters. Thus, a database with bulk ET analyses of all PDB structures is meant to provide a starting point for more detailed analyses, which is made possible by providing direct access to all ET parameters. Still, as is, this integrated web interface will allow other users to quickly determine a baseline generic importance of sequence positions, and often to immediately narrow their search for functional residues to target for mutational analysis of their functional roles or for redesign purposes.

## CONCLUSION

UET complements existing computational and biophysical approaches (32–34) and provides simple and universal access to interpret protein structures and sequences in light of their evolutionary variations and divergences. Unlike simpler measures of residue conservation, ET explicitly correlates evolutionary substitutions with functional divergences estimated by evolutionary distances. This explicit coupling between sequence variations and fitness variations means that ET is best interpreted as a formal gradient of the evolutionary function between genotype and phenotype in the fitness landscape, an observation with important consequences (5,15,35,36). The fundamental role of this evolutionary gradient explains the myriad uses of ET in guiding predictions and rational engineering of protein functional sites, activity and binding.
